# Application and insights on patient-based real-time quality control: detecting undetected errors in internal quality control through daily antibody positivity rate analysis

**DOI:** 10.11613/BM.2025.020801

**Published:** 2025-04-15

**Authors:** Chaochao Ma, Qi Zhang, Yingying Hu, Wenyi Ding, Liangyu Xia, Ling Qiu

**Affiliations:** 1 Department of Laboratory Medicine, Peking Union Medical College Hospital, Peking Union Medical College & Chinese Academy of Medical Science, Beijing, PR China; 2 Department of Occupational and Environmental Health Sciences, School of Public Health, Peking University, Beijing, PR China; 3 State Key Laboratory of Complex Severe and Rare Diseases, Peking Union Medical College Hospital, Peking Union Medical College & Chinese Academy of Medical Science, Beijing, PR China

**Keywords:** Patient-Based Real-Time Quality Control, daily positivity rate, thyroid peroxidase antibody, systematic error, quality assessment

## Abstract

**Introduction:**

Traditional internal quality control (IQC) has limitations in detecting systematic errors in clinical laboratories. Patient-Based Real-Time Quality Control (PBRTQC) has emerged as a complementary method, offering new approaches for quality monitoring. Among these, monitoring daily positivity rates provides meaningful insights into laboratory performance.

**Materials and methods:**

This study highlights a case in which PBRTQC was implemented to detect and address a reagent batch issue in thyroid peroxidase antibody (TPO-Ab) testing. Over one year (July 2023 to July 2024), daily positivity rates and their fluctuations were retrospectively analyzed and daily positivity rate alarm limits were established for monitoring.

**Results:**

A notable increase in the TPO-Ab positivity rate was identified starting in June 2024. For outpatients and inpatients, the positivity rates in June and July 2024 were 46.1% ± 7.8% (N = 9039) and 61.4% ± 12.0% (N = 8735), respectively. For the physical examination population, the positivity rates during the same months were 30.0% ± 11.7% (N = 4754) and 52.5% ± 18.1% (N = 5726), respectively. These rates were significantly higher than the pre-June 2024 average monthly positivity rates of 30.0% ± 2.9% (N = 9070 *per* month) for patients and 11.0% ± 2.4% (N = 4663 *per* month) for the physical examination population.

**Conclusions:**

PBRTQC, particularly monitoring daily positivity rates, is a valuable tool for early detection of systematic errors. Establishing PBRTQC systems can supplement traditional IQC to improve laboratory test quality.

## Graphical abstract

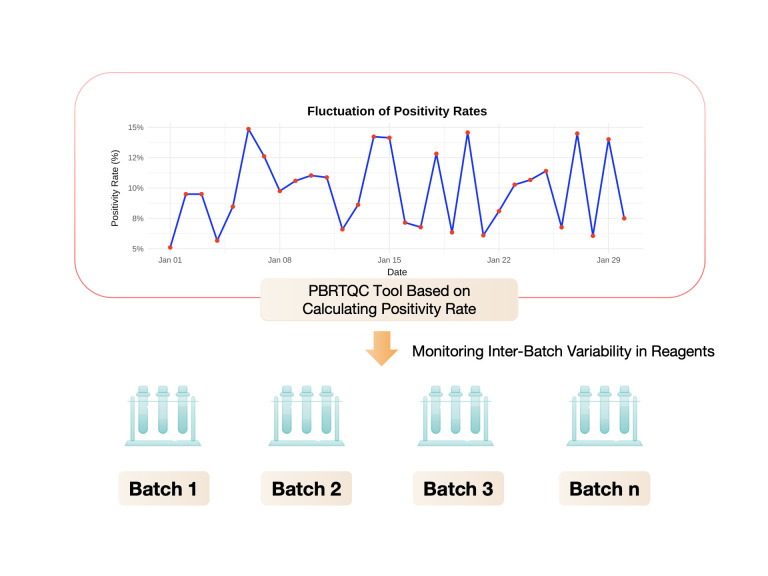

## Highlights

• Introduced daily positivity rate as a novel Patient-Based Real-Time Quality Control (PBRTQC) approach for handling skewed data

• Case study showed how daily positivity rate monitoring identified and resolved a quality issue missed by traditional internal quality control methods

• Advocates integrating PBRTQC into routine workflows to enhance early detection of reagent variations and reduce systematic errors

## Introduction

To address the limitations of traditional internal quality control (IQC), clinical laboratories have increasingly adopted and researched Patient-Based Real-Time Quality Control (PBRTQC) in recent years. Van Rossum and colleagues have significantly contributed to the development and validation of PBRTQC processes (*1*-*3*). New algorithms and evaluation methods for PBRTQC continue to emerge, enhancing its ability to accurately and rapidly detect systematic errors (*4*, *5*). However, when dealing with highly right-skewed data, smoothing techniques such as moving average, weighted moving average, or exponentially weighted moving average often yield suboptimal results. The moving quantile method, while useful, can produce significant fluctuations within the moving window that may lack practical significance. In contrast, the daily positivity rate of test items offers meaningful insights, with relatively low variability when calculated on a daily basis. As a form of PBRTQC, monitoring daily positivity rates can serve as an effective tool for assessing test quality. Although not a real-time monitoring tool, it provides a retrospective analysis approach by calculating relevant quality indicators, such as weekly average positivity rates and standard deviations, to assess the quality of laboratory tests and identify issues that traditional IQC may overlook. The following is a brief study from our laboratory demonstrating the application of PBRTQC and offering quality assessment based on this example.

## Materials and methods

From July 2023 to July 2024, four reagent batches were used in thyroid peroxidase antibody (TPO-Ab) testing in our laboratory. For each batch change, when the stock of the current reagent batch is nearly exhausted, we order and receive a new batch of reagents. Subsequently, we load both the new and old batches of reagents on the same analyzer and test the TPO-Ab concentrations in the same set of specimens from 10 patients, ensuring the comparability of results across batches. The specific calculation method involves determining the differences between the results obtained with the new and old reagent batches. These differences are compared to half of the allowable total error (TEa). If more than 2 out of 10 differences exceed half of the TEa, the batch comparison is deemed unacceptable. Internal quality control is conducted twice daily, using two levels of quality control materials. The 13s, 22s, and R4s rules are applied and considered as out-of-control criteria (*6*). Internal quality control procedures confirmed that the system was under control with each new batch.

To monitor quality, daily positivity rates were calculated for outpatients and inpatients, and the physical examination population. The physical examination population primarily consists of individuals from the community undergoing routine health checks, most of whom are apparently healthy. The positivity rate for TPO-Ab is defined as the proportion of samples with TPO-Ab concentrations greater than or equal to 60 IU/mL relative to the total number of patients tested for TPO-Ab on that specific day. The data is extracted through the Laboratory Information System. The alarm limits are calculated as the mean ± 3 standard deviations based on data collected from July 2023 to March 2024, as this dataset exhibits small and regular fluctuations. The standard deviation of the daily TPO-Ab positivity rates for each month is less than 4.0%. Data analysis and visualization were performed using R (version 4.3.1), with the figures created using the ggplot2 package (version 3.5.1) (*7*, *8*).

## Results

A significant increase in the TPO-Ab positivity rate was observed starting in June 2024. Specifically, for outpatients and inpatients, the positivity rates in June and July 2024 were 46.1% ± 7.8% (N = 9039) and 61.4% ± 12.0% (N = 8735), respectively. For the physical examination population, the positivity rates during the same months were 30.0% ± 11.7% (N = 4754) and 52.5% ± 18.1% (N = 5726), respectively. These rates were significantly higher than the pre-June 2024 average monthly positivity rates of 30.0% ± 2.9% (N = 9070 per month) for patients and 11.0% ± 2.4% (N = 4663 per month) for the physical examination population. During this period, internal quality control did not indicate any systematic bias.

**Figure 1:**
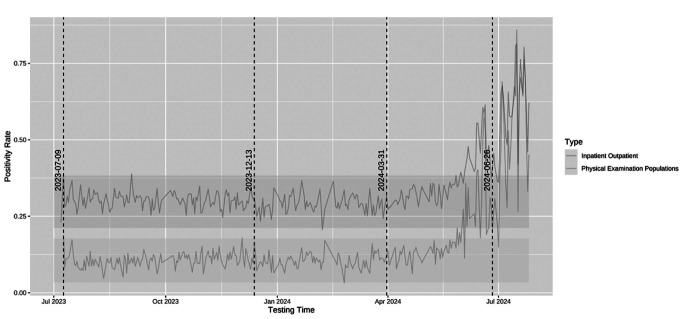
Daily positivity rate fluctuations. This figure illustrates the daily fluctuations in positivity rates of thyroid peroxidase antibody (TPO-Ab) testing. The four vertical lines in the graph represent the time points when the batch numbers were changed. The orange shaded area indicates the alarm limits for the outpatient/inpatient population (0.212 to 0.383), and the cyan shaded area indicates the alarm limits for the physical examination population (0.034 to 0.178). The physical examination population primarily consists of individuals from the community undergoing routine health checks, most of whom are apparently healthy. These alarm limits are calculated as the mean ± 3 standard deviations based on data collected from July 2023 to March 2024, as this dataset exhibits small and regular fluctuations with the standard deviation of the daily TPO-Ab positivity rates for each month being less than 4.0%.

Retrospective analysis of daily positivity rate fluctuations (Figure 1) revealed frequent increases in the daily positivity rate starting after June 1. Further investigation revealed quality issues with the reagent batch, including small, difficult-to-detect clots and significant inter-vial variability. These issues were not identified by IQC but were highlighted through PBRTQC monitoring. The affected batch was replaced, reports recalled, and clinical risks mitigated through retesting and communication with stakeholders.

## Discussion

This experience with PBRTQC using real-world patient data has provided valuable insights into its application and benefits in clinical laboratories (*9*, *10*). First, PBRTQC proves to be an excellent tool for quality control. For laboratories that lack the platform or capability to fully implement PBRTQC, monitoring daily positivity rates and observing their fluctuations and variability can serve as an effective supplementary method for quality assessment. While reagent comparisons are typically conducted following changes in batch numbers and may pass individually, a consistent trend of higher or lower values in every new batch compared to the previous one can result in the accumulation of systematic errors over time. This gradual accumulation of errors can easily be overlooked by laboratory personnel. Therefore, for this particular situation, the comparison did not provide an early indication of the problem with the new reagent batch. By using this PBRTQC method, laboratories can detect batch-to-batch variation in reagents early, thereby mitigating the potential accumulation of these errors.

Furthermore, a PBRTQC system based on patient data can be established by defining specific indicators such as weekly positivity rates and standard deviations. These metrics enable laboratories to evaluate the presence of systematic errors in test results and assess the variability between reagent batches. For instance, a sudden increase in the standard deviation for a specific reagent batch could indicate heightened inter-batch variability, warranting immediate investigation and corrective action. Such a system not only enhances the precision of quality control but also ensures the reliability of laboratory results.

While much research has been dedicated to algorithms and validation processes for establishing PBRTQC, sharing practical applications and case studies, such as the one presented here, is equally crucial. These real-world examples offer meaningful insights and practical guidance for the day-to-day operations of clinical laboratories.

By discussing case-based methodologies, laboratories can better understand the challenges and solutions associated with implementing PBRTQC. Looking forward, we plan to compile similar cases and publish related articles to serve as a reference for industry professionals, further supporting the adoption and refinement of PBRTQC systems in clinical settings.

## Data Availability

The data generated and analyzed in the presented study are available from the corresponding author on request.
